# 1-Bromo­methyl-1,4-diazo­niabicyclo­[2.2.2]octane tetra­chloridozincate

**DOI:** 10.1107/S1600536811032430

**Published:** 2011-08-27

**Authors:** Ping Ping Shi

**Affiliations:** a Ordered Matter Science Research Center, College of Chemistry and Chemical Engineering, Southeast University, Nanjing 211189, People’s Republic of China

## Abstract

The reaction of 1-bromo­methyl-1,4-diazo­niabicyclo­[2.2.2]octane bromide, zinc chloride and hydro­chloric acid in water yields the title compound, (C_7_H_15_BrN_2_)[ZnCl_4_]. In the crystal, the components are linked by N—H⋯Cl hydrogen bonds. The Zn^II^ atom has an approximately tetra­hedral coordination geometry.

## Related literature

For applications of ferroelectric materials, see: Fu *et al.* (2009 ▶); Ye *et al.* (2009 ▶); Zhang *et al.* (2009 ▶). 1,4-diazo­niabicyclo­[2.2.2]octane (DABCO) salts with inorganic tetra­hedral anions exhibit exceptional properties, see: Szafrański *et al.* (2002 ▶). Furthermore, DABCO can undergo substitution with dibromo­methane to obtain 1-bromo­methyl-DABCO bromide, see: Finke *et al.* (2010 ▶).
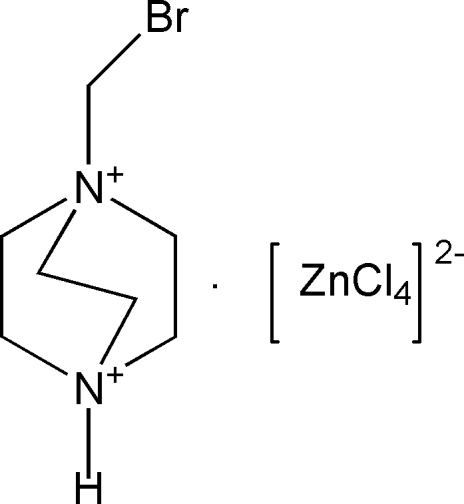


## Experimental

### 

#### Crystal data

(C_7_H_15_BrN_2_)[ZnCl_4_]
*M* *_r_* = 414.30
Monoclinic, 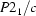

*a* = 10.253 (2) Å
*b* = 12.214 (2) Å
*c* = 11.147 (2) Å
β = 90.97 (3)°
*V* = 1395.7 (4) Å^3^
*Z* = 4
Mo *K*α radiation
μ = 5.36 mm^−1^
*T* = 293 K
0.20 × 0.20 × 0.20 mm

#### Data collection

Rigaku SCXmini diffractometer
Absorption correction: multi-scan (*CrystalClear*; Rigaku, 2005 ▶) *T*_min_ = 0.342, *T*_max_ = 0.356
14183 measured reflections
3201 independent reflections
2698 reflections with *I* > 2σ(*I*)
*R*_int_ = 0.040

#### Refinement

*R*[*F* ^2^ > 2σ(*F* ^2^)] = 0.041
*wR*(*F* ^2^) = 0.099
*S* = 1.12
3201 reflections
136 parameters
H-atom parameters constrained
Δρ_max_ = 1.12 e Å^−3^
Δρ_min_ = −0.92 e Å^−3^

### 

Data collection: *CrystalClear* (Rigaku, 2005 ▶); cell refinement: *CrystalClear*; data reduction: *CrystalClear*; program(s) used to solve structure: *SHELXS97* (Sheldrick, 2008 ▶); program(s) used to refine structure: *SHELXL97* (Sheldrick, 2008 ▶); molecular graphics: *SHELXTL* (Sheldrick, 2008) ▶; software used to prepare material for publication: *SHELXL97*.

## Supplementary Material

Crystal structure: contains datablock(s) I, global. DOI: 10.1107/S1600536811032430/qm2022sup1.cif

Structure factors: contains datablock(s) I. DOI: 10.1107/S1600536811032430/qm2022Isup2.hkl

Additional supplementary materials:  crystallographic information; 3D view; checkCIF report

## Figures and Tables

**Table 1:** Hydrogen-bond geometry (Å, °)

*D*—H⋯*A*	*D*—H	H⋯*A*	*D*⋯*A*	*D*—H⋯*A*
N2—H2*C*⋯Cl2^i^	0.91	2.64	3.313 (4)	131
N2—H2*C*⋯Cl1^i^	0.91	2.75	3.405 (4)	130
N2—H2*C*⋯Cl4^ii^	0.91	2.82	3.363 (3)	120

Symmetry codes: (i) 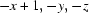; (ii) 
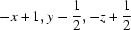
.

**Fig. 1.**
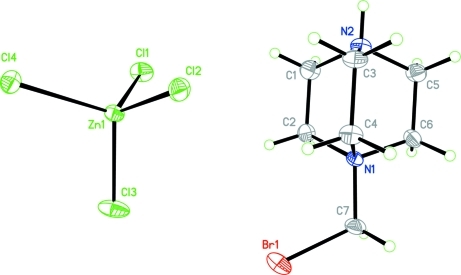
The molecule structure of the title compound,with atom lables and 30% probability displacement ellipsoids for non-H atoms.

**Fig. 2.**
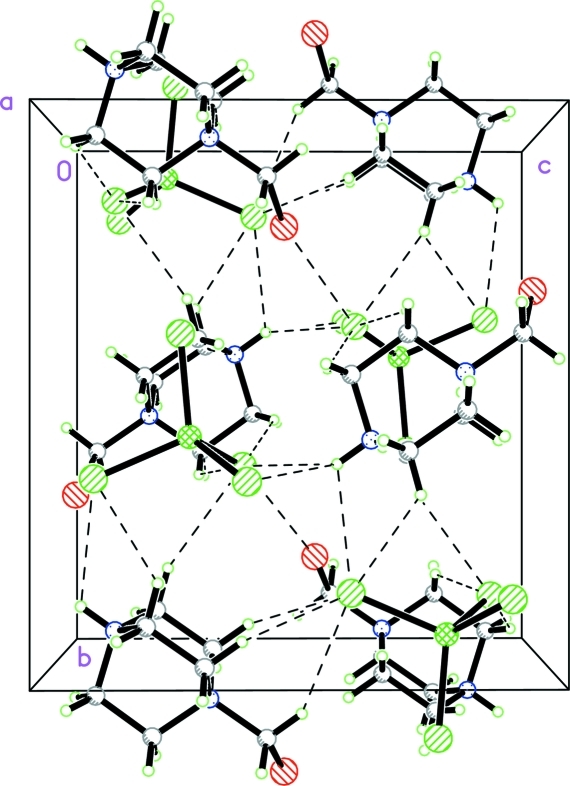
The packing of the title compound,showing molecules connected by N—H···Cl hydrogen bonds (dash lines).

## supplementary crystallographic information

### Comment

Ferroelectric materials have so many potential applications in memory storage that they attract much attention(Fu *et al.*, 2009; Ye *et al.*, 2009; Zhang *et al.*, 2009). In order to find more dielectric or ferroelectric materials, many novel compounds have been synthesized. Thereinto 1,4-diazoniabicyclo[2.2.2]octane (DABCO) salts with inorganic tetrahedral anions exhibit exceptional properties (Szafrański *et al.*,2002). Furthermore, DABCO can undergo substitution with dibromomethane to obtain 1-Bromomethyl-DABCO bromide (Finke *et al.*,2010).

Therefore, we report the single-crystal structure of the title copound which consists of a 1-Bromomethyl-1,4-diazoniabicyclo[2.2.2]octane-1,4-diium cation and a tetrachloridozincate dianion(Fig.1).In the ctystal structure,as showed in the packing diagram(Fig.2), the protonated N2 atom of DABCO derivant interacts *via* a trifurcated hydrogen bond with three Cl atoms of the two neighbouring anions.

However, within the measured temperature range from 190 K to near its melting point(m.p. > 473 K), the dielectric constant of the title compound is basically temperature-independant, suggesting that this material should be not a real ferroelectric.

### Experimental

The mixture solution of 1,4-diazoniabicyclo[2.2.2]octane (20 mmol,2.24 g) and dibromomethane (20 mmol,3.48 g) in acetone was stirred for three hours. A white precipite of 1-Bromomethyl-1,4-diazoniabicyclo[2.2.2]octane-1-ium bromide(1) was synthesized. At room temperature,by slow evaporation of a hydrochloric acid solution containing 1 (20 mmol,5.72 g) and zinc chloride (20 mmol,2.72 g),colorless crystals of the title copound suitable for X-ray analysis were obtained.

### Refinement

All H atoms were positioned geometrically with C—H = 0.97 Å, *N*—H = 0.91 Å, and refined using a riding model, with *U*_iso_(H) = 1.2_eq_(C,*N*).

### Crystal data

**Table:**

(C_7_H_15_BrN_2_)[ZnCl_4_]	*F*(000) = 816
*M**_r_* = 414.30	*D*_x_ = 1.972 Mg m^−^^3^
Monoclinic, *P*2_1_/*c*	Mo *K*α radiation, λ = 0.71073 Å
Hall symbol: -P 2ybc	Cell parameters from 12777 reflections
*a* = 10.253 (2) Å	θ = 3.2–27.5°
*b* = 12.214 (2) Å	µ = 5.36 mm^−^^1^
*c* = 11.147 (2) Å	*T* = 293 K
β = 90.97 (3)°	Prism, colorless
*V* = 1395.7 (4) Å^3^	0.20 × 0.20 × 0.20 mm
*Z* = 4	

### Data collection

**Table:**

Rigaku SCXmini diffractometer	3201 independent reflections
Radiation source: fine-focus sealed tube	2698 reflections with *I* > 2σ(*I*)
graphite	*R*_int_ = 0.040
Detector resolution: 13.6612 pixels mm^-1^	θ_max_ = 27.5°, θ_min_ = 3.2°
CCD_Profile_fitting scans	*h* = −13→13
Absorption correction: multi-scan (*CrystalClear*; Rigaku, 2005)	*k* = −15→15
*T*_min_ = 0.342, *T*_max_ = 0.356	*l* = −14→14
14183 measured reflections	

### Refinement

**Table:**

Refinement on *F*^2^	Primary atom site location: structure-invariant direct methods
Least-squares matrix: full	Secondary atom site location: difference Fourier map
*R*[*F*^2^ > 2σ(*F*^2^)] = 0.041	Hydrogen site location: inferred from neighbouring sites
*wR*(*F*^2^) = 0.099	H-atom parameters constrained
*S* = 1.12	*w* = 1/[σ^2^(*F*_o_^2^) + (0.0381*P*)^2^ + 2.7437*P*] where *P* = (*F*_o_^2^ + 2*F*_c_^2^)/3
3201 reflections	(Δ/σ)_max_ < 0.001
136 parameters	Δρ_max_ = 1.12 e Å^−^^3^
0 restraints	Δρ_min_ = −0.92 e Å^−^^3^

### Special details

**Table:**

Geometry. All e.s.d.'s (except the e.s.d. in the dihedral angle between two l.s. planes) are estimated using the full covariance matrix. The cell e.s.d.'s are taken into account individually in the estimation of e.s.d.'s in distances, angles and torsion angles; correlations between e.s.d.'s in cell parameters are only used when they are defined by crystal symmetry. An approximate (isotropic) treatment of cell e.s.d.'s is used for estimating e.s.d.'s involving l.s. planes.
Refinement. Refinement of *F*^2^ against ALL reflections. The weighted *R*-factor *wR* and goodness of fit *S* are based on *F*^2^, conventional *R*-factors *R* are based on *F*, with *F* set to zero for negative *F*^2^. The threshold expression of *F*^2^ > σ(*F*^2^) is used only for calculating *R*-factors(gt) *etc*. and is not relevant to the choice of reflections for refinement. *R*-factors based on *F*^2^ are statistically about twice as large as those based on *F*, and *R*- factors based on ALL data will be even larger.

### Fractional atomic coordinates and isotropic or equivalent isotropic displacement parameters (Å^2^)

**Table:**

	*x*	*y*	*z*	*U*_iso_*/*U*_eq_	
Zn1	0.22858 (4)	0.07443 (4)	0.21406 (4)	0.02816 (13)	
Br1	0.60658 (5)	0.19087 (4)	0.46852 (4)	0.04538 (15)	
Cl1	0.06383 (9)	0.14865 (9)	0.09911 (9)	0.0345 (2)	
Cl2	0.41010 (10)	0.13065 (10)	0.11498 (9)	0.0379 (2)	
Cl3	0.21448 (13)	−0.10851 (8)	0.23132 (11)	0.0455 (3)	
Cl4	0.22216 (16)	0.14841 (9)	0.40008 (9)	0.0534 (4)	
N1	0.7521 (3)	0.0408 (2)	0.3236 (3)	0.0220 (6)	
C5	0.7422 (4)	0.1103 (3)	0.2117 (3)	0.0294 (8)	
H5A	0.8157	0.1601	0.2089	0.035*	
H5B	0.6628	0.1534	0.2130	0.035*	
N2	0.7501 (3)	−0.0797 (3)	0.1395 (3)	0.0299 (7)	
H2C	0.7495	−0.1232	0.0732	0.036*	
C2	0.6364 (4)	−0.1081 (4)	0.2143 (4)	0.0383 (10)	
H2A	0.6409	−0.1847	0.2371	0.046*	
H2B	0.5562	−0.0967	0.1686	0.046*	
C4	0.8766 (4)	−0.0259 (4)	0.3192 (4)	0.0313 (9)	
H4A	0.8840	−0.0717	0.3900	0.038*	
H4B	0.9514	0.0228	0.3181	0.038*	
C6	0.7411 (4)	0.0367 (3)	0.1011 (3)	0.0316 (9)	
H6A	0.6614	0.0482	0.0548	0.038*	
H6B	0.8143	0.0548	0.0508	0.038*	
C3	0.8752 (4)	−0.0969 (4)	0.2076 (4)	0.0371 (10)	
H3A	0.9483	−0.0780	0.1576	0.045*	
H3B	0.8836	−0.1733	0.2302	0.045*	
C1	0.6368 (4)	−0.0367 (3)	0.3260 (4)	0.0315 (9)	
H1A	0.5564	0.0049	0.3290	0.038*	
H1B	0.6425	−0.0824	0.3971	0.038*	
C7	0.7626 (4)	0.1111 (4)	0.4357 (3)	0.0341 (9)	
H7A	0.8338	0.1626	0.4267	0.041*	
H7B	0.7835	0.0645	0.5038	0.041*	

### Atomic displacement parameters (Å^2^)

**Table:**

	*U*^11^	*U*^22^	*U*^33^	*U*^12^	*U*^13^	*U*^23^
Zn1	0.0349 (3)	0.0251 (2)	0.0246 (2)	−0.00060 (18)	0.00461 (18)	0.00046 (18)
Br1	0.0530 (3)	0.0438 (3)	0.0399 (3)	0.0122 (2)	0.0152 (2)	−0.0048 (2)
Cl1	0.0290 (5)	0.0422 (6)	0.0323 (5)	−0.0009 (4)	0.0019 (4)	0.0055 (4)
Cl2	0.0273 (5)	0.0515 (6)	0.0351 (5)	−0.0061 (4)	0.0039 (4)	−0.0019 (5)
Cl3	0.0708 (8)	0.0177 (5)	0.0478 (6)	0.0032 (5)	−0.0015 (6)	0.0023 (4)
Cl4	0.1096 (11)	0.0293 (5)	0.0214 (5)	0.0050 (6)	0.0062 (6)	0.0014 (4)
N1	0.0254 (15)	0.0230 (15)	0.0178 (14)	0.0006 (12)	0.0041 (12)	0.0003 (12)
C5	0.041 (2)	0.0245 (19)	0.0231 (19)	−0.0007 (16)	0.0025 (17)	0.0050 (15)
N2	0.0380 (19)	0.0261 (17)	0.0258 (16)	0.0002 (14)	0.0020 (14)	−0.0046 (13)
C2	0.041 (2)	0.031 (2)	0.043 (2)	−0.0126 (19)	0.008 (2)	−0.0026 (19)
C4	0.030 (2)	0.036 (2)	0.028 (2)	0.0072 (17)	0.0029 (16)	−0.0013 (17)
C6	0.045 (2)	0.029 (2)	0.0209 (19)	0.0009 (18)	0.0001 (17)	−0.0003 (16)
C3	0.038 (2)	0.034 (2)	0.039 (2)	0.0142 (18)	−0.0051 (19)	−0.0089 (19)
C1	0.030 (2)	0.034 (2)	0.031 (2)	−0.0063 (17)	0.0062 (16)	0.0022 (17)
C7	0.040 (2)	0.040 (2)	0.0229 (19)	0.0052 (18)	0.0022 (17)	−0.0085 (17)

### Geometric parameters (Å, °)

**Table:**

Zn1—Cl3	2.2475 (12)	N2—H2C	0.9100
Zn1—Cl4	2.2639 (12)	C2—C1	1.521 (6)
Zn1—Cl2	2.2858 (12)	C2—H2A	0.9700
Zn1—Cl1	2.2899 (12)	C2—H2B	0.9700
Br1—C7	1.913 (4)	C4—C3	1.516 (5)
N1—C5	1.511 (5)	C4—H4A	0.9700
N1—C1	1.515 (5)	C4—H4B	0.9700
N1—C4	1.516 (5)	C6—H6A	0.9700
N1—C7	1.519 (5)	C6—H6B	0.9700
C5—C6	1.525 (5)	C3—H3A	0.9700
C5—H5A	0.9700	C3—H3B	0.9700
C5—H5B	0.9700	C1—H1A	0.9700
N2—C2	1.486 (5)	C1—H1B	0.9700
N2—C6	1.487 (5)	C7—H7A	0.9700
N2—C3	1.494 (5)	C7—H7B	0.9700
			
Cl3—Zn1—Cl4	108.39 (5)	N1—C4—C3	109.8 (3)
Cl3—Zn1—Cl2	113.21 (5)	N1—C4—H4A	109.7
Cl4—Zn1—Cl2	111.05 (5)	C3—C4—H4A	109.7
Cl3—Zn1—Cl1	113.17 (5)	N1—C4—H4B	109.7
Cl4—Zn1—Cl1	108.78 (5)	C3—C4—H4B	109.7
Cl2—Zn1—Cl1	102.10 (4)	H4A—C4—H4B	108.2
C5—N1—C1	108.9 (3)	N2—C6—C5	109.3 (3)
C5—N1—C4	108.7 (3)	N2—C6—H6A	109.8
C1—N1—C4	108.8 (3)	C5—C6—H6A	109.8
C5—N1—C7	111.4 (3)	N2—C6—H6B	109.8
C1—N1—C7	112.5 (3)	C5—C6—H6B	109.8
C4—N1—C7	106.5 (3)	H6A—C6—H6B	108.3
N1—C5—C6	109.6 (3)	N2—C3—C4	109.4 (3)
N1—C5—H5A	109.7	N2—C3—H3A	109.8
C6—C5—H5A	109.7	C4—C3—H3A	109.8
N1—C5—H5B	109.7	N2—C3—H3B	109.8
C6—C5—H5B	109.7	C4—C3—H3B	109.8
H5A—C5—H5B	108.2	H3A—C3—H3B	108.2
C2—N2—C6	109.8 (3)	N1—C1—C2	109.6 (3)
C2—N2—C3	110.9 (3)	N1—C1—H1A	109.8
C6—N2—C3	109.2 (3)	C2—C1—H1A	109.8
C2—N2—H2C	108.9	N1—C1—H1B	109.8
C6—N2—H2C	108.9	C2—C1—H1B	109.8
C3—N2—H2C	108.9	H1A—C1—H1B	108.2
N2—C2—C1	109.5 (3)	N1—C7—Br1	113.4 (3)
N2—C2—H2A	109.8	N1—C7—H7A	108.9
C1—C2—H2A	109.8	Br1—C7—H7A	108.9
N2—C2—H2B	109.8	N1—C7—H7B	108.9
C1—C2—H2B	109.8	Br1—C7—H7B	108.9
H2A—C2—H2B	108.2	H7A—C7—H7B	107.7

### Hydrogen-bond geometry (Å, °)

**Table:**

*D*—H···*A*	*D*—H	H···*A*	*D*···*A*	*D*—H···*A*
N2—H2C···Cl2^i^	0.91	2.64	3.313 (4)	131.
N2—H2C···Cl1^i^	0.91	2.75	3.405 (4)	130.
N2—H2C···Cl4^ii^	0.91	2.82	3.363 (3)	120.

Symmetry codes: (i) −*x*+1, −*y*, −*z*; (ii) −*x*+1, *y*−1/2, −*z*+1/2.
